# 

**Published:** 1969-04

**Authors:** 


### Contents

| | Page |
|---|---|
| THE CARE OF THE ELDERLY<br>*W. H. Lloyd; A. W. Macara; P. S. Sinclair* | 33 |
| COLDHARBOUR FARM—THE FIRST FIVE YEARS<br>*W. A. Heaton-Ward* | 46 |
| HOST RESISTANCE AND HOSPITAL INFECTION<br>*W. A. Gillespie* | 51 |
| THE CHANGING MEDICAL CURRICULUM | 56 |

